# Targeting hypoxia-inducible factor-1α (HIF-1α) in combination with antiangiogenic therapy: A phase I trial of bortezomib plus bevacizumab

**DOI:** 10.18632/oncotarget.2163

**Published:** 2014-11-08

**Authors:** Gerald S Falchook, Jennifer J Wheler, Aung Naing, Edward F Jackson, Filip Janku, David Hong, Chaan S Ng, Nizar M Tannir, Kristie N Lawhorn, Mei Huang, Laura S Angelo, Deeksha Vishwamitra, Kenneth Hess, Adrienne N Howard, Kristin L Parkhurst, Hesham M Amin, Razelle Kurzrock

**Affiliations:** ^1^ Sarah Cannon Research Institute, Denver, CO 80218; ^2^ Department of Investigational Cancer Therapeutics (Phase I Program), The University of Texas MD Anderson Cancer Center, Houston, TX 77030; ^3^ Department of Medical Physics, University of Wisconsin School of Medicine and Public Health, Madison, WI 53705; ^4^ Department of Diagnostic Radiology, The University of Texas MD Anderson Cancer Center, Houston, TX 77030; ^5^ Department of Genitourinary Medical Oncology, The University of Texas MD Anderson Cancer Center, Houston, TX 77030; ^6^ Department of Head and Neck Surgery, The University of Texas MD Anderson Cancer Center, Houston, TX 77030; ^7^ Department of Immunology, Allergy, and Rheumatology, The Center for Human Immunobiology, Texas Children's Hospital/Baylor College of Medicine, Houston, TX 77030; ^8^ Department of Hematopathology, The University of Texas MD Anderson Cancer Center, Houston, TX 77030; ^9^ Department of Biostatistics, The University of Texas MD Anderson Cancer Center, Houston, TX 77030; ^10^ Center for Personalized Cancer Therapy, Moores Cancer Center, University of California, San Diego, San Diego, CA 92093

**Keywords:** bevacizumab, bortezomib, phase 1, proteasome, HIF-1α

## Abstract

**Purpose:**

We hypothesized that bortezomib, an agent that suppresses HIF-1α transcriptional activity, when combined with bevacizumab, would obviate the HIF-1α resistance pathway. The objectives of this phase I trial were to assess safety and biological activity of this combination.

**Experimental Design:**

Patients with advanced, refractory malignancies were eligible. Patients received bevacizumab and bortezomib (3-week cycle) with dose expansions permitted if responses were seen and for assessing correlates. Pharmacodynamic assessment included plasma VEGF, VEGFR2, 20S proteasome inhibition, dynamic contrast-enhanced magnetic resonance imaging (DCE-MRI), and HIF-1α tumor expression.

**Results:**

Ninety-one patients were treated (median=6 prior treatments). The FDA-approved doses of both drugs were safely reached, and the recommended phase 2 dose (RP2D) is bevacizumab 15 mg/kg with bortezomib 1.3 mg/m^2^. Four patients attained partial response (PR) and seven patients achieved stable disease (SD) ≥6 months (Total SD≥6 months/PR=11 (12%)). The most common drug-related toxicities included thrombocytopenia (23%) and fatigue (19%). DCE-MRI analysis demonstrated no dose-dependent decreases in *K^trans^* although analysis was limited by small sample size (N=12).

**Conclusion:**

Combination bevacizumab and bortezomib is well-tolerated and has demonstrated clinical activity in patients with previously treated advanced malignancy. Pharmacodynamic assessment suggests that inhibition of angiogenic activity was achieved.

## INTRODUCTION

Angiogenesis, which has a fundamental role in tumor growth and metastasis [1-3], is important for supplying a growing tumor with oxygen, nutrients, growth factors, hormones, proteolytic enzymes, and hemolytic factors, and is a critical step in the pathogenesis of metastasis [2-4]. Increased tumor vascularization and tumor expression of pro-angiogenic factors has been associated with advanced tumor stage and poor prognosis [3]. The vascular endothelial growth factor (VEGF) family of proteins and receptors play a pivotal role in tumor angiogenesis and in the pathogenesis of a wide range of human cancers [5].

One mechanism of tumor resistance to anti-angiogenic therapy is upregulation of the transcription factor hypoxia-inducible factor 1α (HIF-1α), which mediates adaptive responses to hypoxic conditions commonly found in solid tumors [3, 6-14]. HIF-1α promotes expression of various genes that encode proteins involved in inflammatory reactions involved in cancer [15]. HIF-1α overexpression has been identified in a number of tumor types, including pancreatic, head and neck, breast, renal, ovarian, bladder, brain, colorectal, and prostate cancers, and overexpression correlates with increased angiogenesis and metastasis [15, 16]. Thus, HIF-1α inhibition in combination with anti-angiogenic therapy is a promising strategy for targeting tumor resistance [11, 17-20].

Anti-angiogenic agents that target the VEGF pathway have demonstrated clinical benefit for a variety of malignancies, including colorectal, lung, glioblastoma, ovarian, and renal cell cancer [3, 10, 21-25]. Bevacizumab is a monoclonal antibody to VEGF that is FDA approved for the treatment of renal cell carcinoma, colorectal cancer, non-small cell lung cancer, and glioblastoma.

The proteasome is a large enzyme complex responsible for the regulation of proteins involved in various cell signaling pathways by degradation [26-28] and proteasome inhibition induces apoptosis by interfering with the regulation of signaling cascades implicated in cancer, such as the nuclear factor NF-κB [27, 29]. Bortezomib, a boronic acid bipeptide, is a specific and reversible inhibitor of the chymotrypsin-like activity of the 26S proteasome [30, 31]. While bortezomib exhibits pro-apoptotic, anti-angiogenic, and anti-proliferative activity, it has also demonstrated the ability to inhibit transcriptional activity of HIF-1α [27, 28, 30-33]. This appears to be a class effect as the proteasome inhibitors bortezomib and NPI-0052 have been shown *in vitro* to inhibit tumor angiogenesis as a result of decreased VEGF expression via downregulation of HIF-1α [33, 34]. Bortezomib is FDA approved for the treatment of multiple myeloma and mantle cell lymphoma. In phase I and II clinical trials, partial responses (PR) have been achieved in various solid tumors, including metastatic or recurrent renal cell carcinoma, non-small cell lung carcinoma, ovarian adenocarcinoma, pancreatic adenocarcinoma, and sarcoma [27, 31].

We performed a phase I trial administering sequential bevacizumab and bortezomib based on our hypothesis that this combination will obviate the HIF-1α pathway as a mechanism of resistance to bevacizumab. The primary objective of this study was to determine the maximum tolerated dose and dose-limiting toxicities of the combination treatment of bevacizumab with bortezomib. The secondary objectives were to establish a preliminary descriptive assessment of anti-tumor efficacy and anti-angiogenesis correlates with the drug combination.

## RESULTS

### Patient Characteristics

Ninety-one patients were enrolled (median 52.5 years old, range 27-78). The median number of prior systemic treatments was six. The majority of patients had an ECOG performance status of 1. The most common tumor types enrolled were RCC, breast cancer, rectal carcinoma, nasopharyngeal, neuroendocrine carcinoma, and prostate cancer (Table 1).

**Table 1:** Patient characteristics

Characteristic	Total
**Number of patients**	91
**Median age**	52.5 yrs (Range 27-78)
**Sex**	
Male	49 (54%)
Female	42 (46%)
**ECOG PS**	
0	7 (8%)
1	74 (81%)
2	10 (11%)
**Prior treatment**	
Surgery	70 (77%)
Radiation	57 (63%)
Chemotherapy	91 (100%)
Phase I trial	34 (37%)
Bevacizumab only	26 (29%)
Bortezomib only	3 (3%)
Bevacizumab and Bortezomib	1 (1%)
**Median number of prior systemic treatments**	5.5 (Range 0-11)
**Median number of prior Phase I treatments**	3 (Range 0-6)
**Diagnosis**	
Renal Cell Carcinoma	21 (23%)
Breast	11 (12%)
Colorectal Carcinoma	11 (12%)
Nasopharyngeal	6 (7%)
Gastric/esophageal	5 (5%)
Neuroendocrine	5 (5%)
Prostate	5 (5%)
Pancreatic	3 (3%)
Melanoma	3 (3%)
Ovarian/fallopian tube	3 (3%)
Leiomyosarcoma	2 (2%)
Hepatocellular	2 (2%)
Cervical	2 (2%)
Urothelial	2 (2%)
Other*	10 (11%)

Abbreviations: ECOG, Eastern Cooperative Oncology Group; PS, performance status; GE, gastroesophageal

* Other tumor types include one of each of the following: adenoid cystic carcinoma, adrenocortical carcinoma, mucinoid carcinoma of the appendix, B-cell lymphoma, cholangiocarcinoma, granular cell carcinoma, paraganglioma, parotid carcinoma, piriform sinus carcinoma, small cell lung carcinoma

### Toxicity and Recommended Dose

All patients were evaluated for toxicity (Table 2). The highest dose level, dose level 9, with doses of bevacizumab at 15mg/kg and bortezomib at 1.3mg/m^2^, was reached without identification of an MTD and further dose escalation was not performed above the FDA-approved doses. Only one DLT was observed, which was grade 4 acute renal failure on Day 19 in a patient on dose level 9.

**Table 2:** Toxicities

Toxicity By Dose Level	DL 1 (N=7)	DL 2 (N=6)	DL 3 (N=6)	DL 4 (N=6)	DL 5 (N=6)	DL 6 (N=6)	DL 7 (N=6)	DL 8 (N=6)	DL 9 (N=37)	Total (N=91)
Bevacizumab mg/kg IV	2.5	2.5	5	5	7.5	7.5	10	12.5	15	
Bortezomib mg/m^2^ IV	0.7 D1, 8	0.7 D1, 4, 8, 11	0.7	1.0	1.0	1.3	1.3	1.3	1.3	
**Thrombocytopenia**									
Grade 2	0	2 (2%)	0	0	0	1 (1%)	3 (3%)	1 (1%)	3 (3%)	10 (11%)
Grade 3	0	0	1 (1%)	0	1 (1%)	1 (1%)	1 (1%)	0	6 (7%)	10 (11%)
Grade 4	0	0	0	0	0	0	1 (1%)	0	0	1 (1%)
**Fatigue**									
Grade 2	1 (1%)	1 (1%)	0	2 (2%)	0	1 (1%)	2 (2%)	1 (1%)	7 (8%)	15 (16%)
Grade 3	0	0	0	0	0	0	0	0	2 (2%)	2 (2%)
**Nausea/Vomiting**									
Grade 2	0	0	0	0	0	1 (1%)	2 (2%)	0	7 (8%)	10 (11%)
Grade 3	0	0	0	0	0	0	0	0	1 (1%)	1 (1%)
**Diarrhea**									
Grade 2	0	0	0	0	0	0	4 (4%)	0	5 (5%)	9 (10%)
Grade 3	0	0	0	0	0	0	0	0	2 (2%)	2 (2%)
**Arthalgia / Myalgia**									
Grade 2	0	0	0	1 (1%)	2 (2%)	1 (1%)	1 (1%)	1 (1%)	3 (3%)	9 (10%)
**Neuropathy**									
Grade 2	1 (1%)	0	0	0	0	0	0	0	6 (7%)	7 (8%)
Grade 3	0	0	0	0	0	0	2 (2%)	0	0	2 (2%)
**Anorexia**										
Grade 2	0	1 (1%)	0	0	0	0	1 (1%)	0	6 (7%)	8 (9%)
**Anemia**									
Grade 2	0	1 (1%)	1 (1%)	1 (1%)	2 (2%)	1 (1%)	0	0	1 (1%)	7 (8%)
Grade 3	0	0	1 (1%)	0	0	0	0	0	0	1 (1%)
**Neutropenia**									
Grade 2	0	0	0	1 (1%)	0	0	1 (1%)	0	0	2 (2%)
Grade 3	0	0	0	0	0	1 (1%)	0	0	2 (2%)	3 (3%)
Grade 4	0	0	0	0	0	0	0	0	1 (1%)	1 (1%)
**Hypertension**									
Grade 2	0	0	0	0	0	0	1 (1%)	0	1 (1%)	2 (2%)
Grade 3	0	0	0	0	0	0	0	3 (3%)	0	3 (3%)

* Grade 3, 4, and 5 events were not included in the table if there were 0 events with that grade.

** Table includes Grade 2 and greater adverse events (AEs) occurring at a frequency of ≥ 5% of participants who received treatment on study. AEs occurring at a rate lower than 5% included: elevated AST/ALT (DL1, Grade 2, n = 1; DL2, Grade 2, n = 1; DL5, Grade 2, n = 1; DL6, Grade 2, n = 1), constipation (DL8, Grade 2, n = 1; DL9, Grade 2, n = 2), proteinuria (DL1, Grade 3, n = 1; DL2, Grade 3, n = 1; DL9, Grade 2, n = 1), pulmonary embolism (DL2, Grade 4, n =1; DL4, Grade 4, n = 1; DL9, Grade 5, n = 1), hemoptysis (DL2, Grade 3, n = 1; DL7, Grade 2, n = 1), elevated bilirubin (DL6, Grade 2, n = 1), dyspnea (DL7, Grade 2, n = 1), GI bleed (DL2, Grade 3, n = 1), headache (DL9, Grade 2, n = 1), hematuria (DL2, Grade 2, n = 1), hypotension (DL9, Grade 2, n = 1), pancreatitis (DL8, Grade 2, n = 1), acute renal failure (DL9, Grade 4, n = 1).

All dose levels were tested and shown to be safe. Overall, the most common drug-related toxicities grade 2 or higher included thrombocytopenia (23%), fatigue (19%), nausea/vomiting (12%), diarrhea (12%), arthralgia/myalgia (10%), anorexia (9%), anemia (9%), neutropenia (7%), and hypertension (6%). Thirty-three patients (36%) experienced no drug-related toxicity higher than grade 1 and 66 patients (73%) had no drug-related toxicity higher than grade 2.

Adverse events that required a dose reduction occurred in 10 patients (11%). The causes of dose reduction were thrombocytopenia (n=5) and neuropathy (n=5). Four patients died while on treatment (two from sepsis, one from acute myocardial infarction likely related to sepsis, one from carotid hemorrhage, and one from suspected pulmonary embolism), but these events were not treatment related. Seven patients were withdrawn due to toxicity, including neuropathy (n=1), neuropathy and gastrointestinal bleed (n=1), pulmonary embolism (n=1), hyponatremia (n=1), renal failure (n=1), renal failure and altered mental status (n=1), and diarrhea, nausea, anorexia and body aches (n=1). Because of adequate safety observed, the recommended phase 2 dose was determined to be level 9, which includes the recommended FDA-approved full dose of each medication, bevacizumab 15mg/kg and bortezomib 1.3mg/m^2^.

### Antitumor Activity

**Figure 1:**
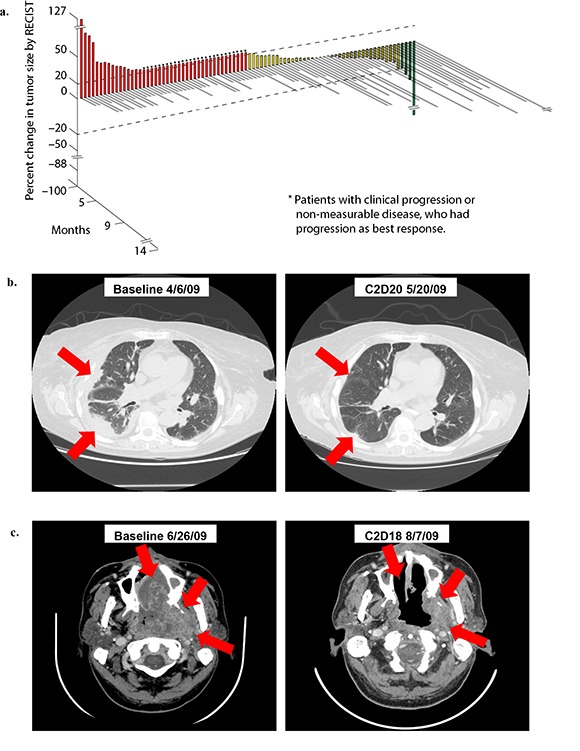
**(a). 3D Waterfall plot showing best response and time on study in 86 evaluable patients.** Five patients omitted from the figure were not evaluable because treatment was discontinued before the first restaging evaluation (one for toxicity (carotid hemorrhage)), and three withdrew consent (one due to side effects and desire to pursue therapy closer to home, one so other therapy could be pursued closer to home, and one because the patient felt the dose of bevacizumab was too low since higher doses were received on previous therapy). The x-axis represents each patient. The y-axis indicates percent change in tumor size by RECIST. Patients who experienced partial response are shown in green, patients with stable disease in yellow, and patients with progressive disease in red. Patients with early clinical progression or new lesions are indicated arbitrarily as +21% and are denoted with a star (*). The treatment duration (months) for each patient is depicted by the grey bars on the z-axis. Bars that are not to scale are denoted with double line “breaks”. **(b). Treatment response of 64 y/o woman with metastatic renal cell carcinoma (RCC).** Patient received 20 cycles of treatment (14 months) and achieved partial response (PR) (88% decrease in tumor size per RECIST). **(c). Treatment response of 48 y/o man with metastatic nasopharyngeal carcinoma.** Patient received 6 cycles of treatment (5 months) and achieved partial response (PR) (38% decrease in tumor size per RECIST). Abbreviations: RECIST, response evaluation criteria in solid tumors

Eleven out of 91 patients (12%) received more than 6 months of treatment (Table 3). Among the 91 treated patients, four patients achieved partial response, 39 patients achieved stable disease (SD), 43 patients had progressive disease (PD), and five patients were inevaluable (Figure 1a). The partial responses included three patients with renal cell carcinoma (−88%, −45%, −30%) (Figure 1b) and one patient with nasopharyngeal carcinoma (−38%) (Figure 1c).

**Table 3:** Patients on study ≥ 6 months or with partial response

Best Response (%)	Treatment duration (months)	Tumor type	Prior bevacizumab	Prior bortezomib	Brain mets	Dose Level	Hypertension grade	VEGF SNPs				Maximum % change in VEGF	Maximum % change in VEGFR2
								2578	1154	1498	634		
−88%	14	RCC	Y	N	N	9	None	A/C	G/A	C/T	C/G	-	-
−45%	8	RCC	N	N	N	2	1	A/C	G/A	C/T	C/G	-	-
−38%	5	NPC	N	Y	N	9	None	-	-	-	-	-	-
−30%	6	RCC	N	N	N	7	2	-	-	-	-	+342%	+10%
−19%	9	Leiomyosarcoma	Y	N	N	4	1	A/C	**AA**	**TT**	C/G	+259%	+3.1%
−15%	8	Fallopian tube	N	N	N	9	1	**AA**	**AA**	CC	GG	+826%	+10.9%
−12%	7	NPC	N	N	N	9	None	-	-	-	-	-	-
−8%	8	Neuroendocrine	N	N	N	6	1	A/C	G/A	C/T	C/G	+1213%	+11.6%
−2%	6	Hepatocellular	Y	Y	N	5	1	A/C	G/A	C/T	C/G	+1566%	-
−2%	7	RCC	N	N	N	7	None	A/C	G/A	C/T	GG	+255%	+8.1%
3%	6	RCC	N	N	Y	9	None	-	-	-	-	-	-

RCC = Renal Cell Carcinoma, NPC = Nasopharyngeal Carcinoma, “-” = Not available

**Figure 2:**
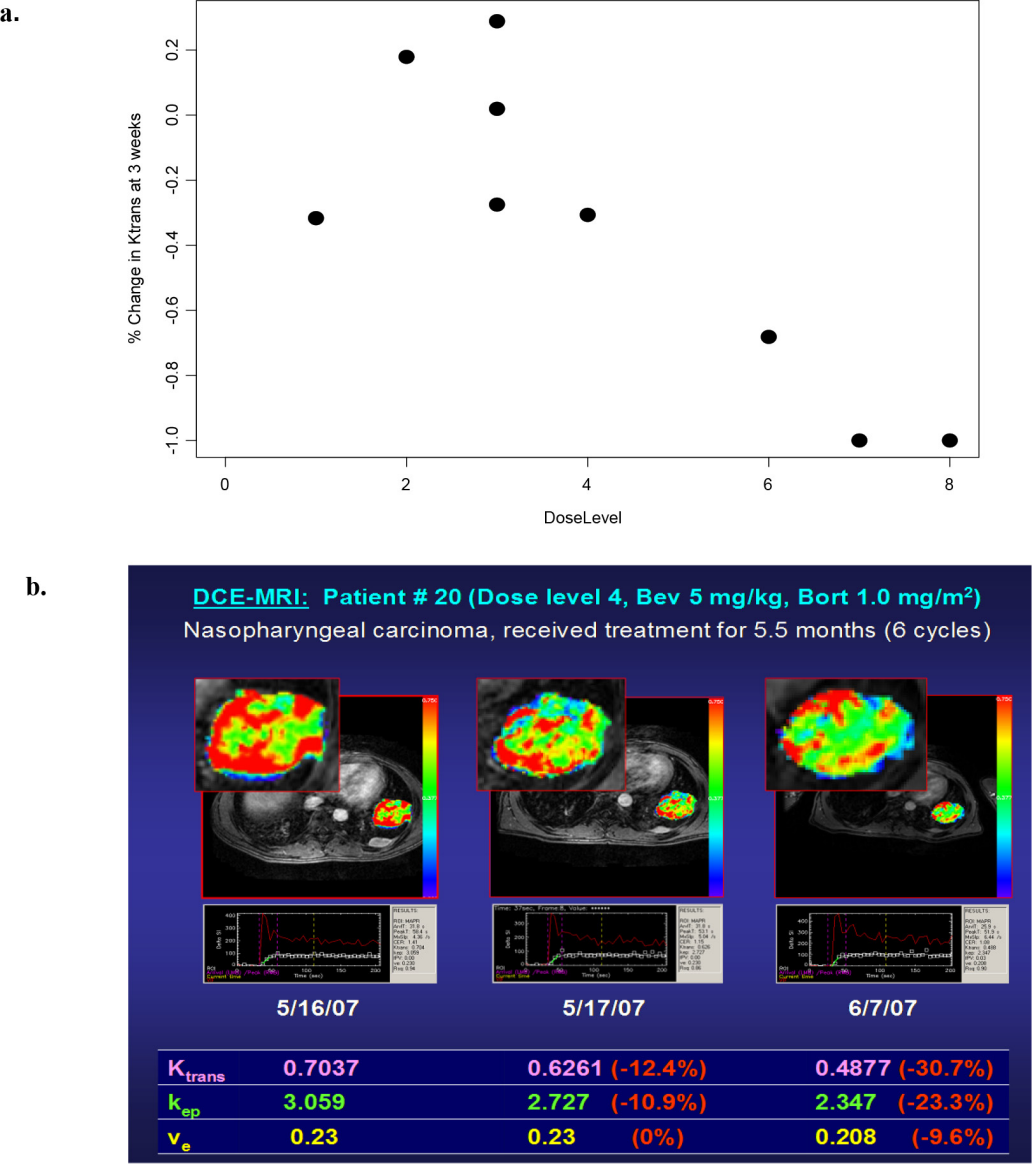
(a). Dose-dependent changes in the volume transfer constant (*Ktrans*) at 3 weeks (n=9) (stratified by bevacizumab dose) (R=−0.83, p=0.0053). (b). DCE-MRI analysis of nasopharyngeal carcinoma On the parametric color map, areas with the highest tissue permeability (as assessed by *Ktrans*) are visualized as red, and areas with the lowest *Ktrans* values are visualized as blue

The median number of months of treatment received was 2 (range 0-14). Sixty-eight out of 91 patients were measurable per RECIST. Among the remaining 23 patients, 18 were evaluable but not measurable and five were not evaluable because treatment was discontinued before the first restaging evaluation (one for toxicity (carotid hemorrhage)), and three withdrew consent (one due to side effects and desire to pursue therapy closer to home, one so other therapy could be pursued closer to home, and one because the patient felt the dose of bevacizumab was too low since higher doses were received on previous therapy). The median time to treatment failure (TTF) was 2 months (range 0-14), and the median overall survival (OS) was 7 months (range 0-43).

### Prior Bevacizumab and/or Bortezomib

Out of 91 patients treated on study, thirty received prior bevacizumab or bortezomib (33%, Table 1). Twenty-six patients received prior bevacizumab only, three patients received prior bortezomib only, and 1 patient received prior bortezomib and bevacizumab, but not concurrently. The median number of months of prior bevacizumab/bortezomib therapy was 4.5. No statistically significant correlation was observed in response or time on treatment when comparing patients who received prior bevacizumab or bortezomib with patients who had not received prior bevacizumab or bortezomib (30 and 61 patients, p=0.43, p=0.73, respectively). Of note, 4 of the 11 patients with SD≥6 months or PR received prior bevacizumab or bortezomib (renal cell carcinoma, nasopharyngeal carcinoma, leiomyosarcoma, hepatocellular carcinoma). The renal cell carcinoma patient with the best response on study (88% decrease in tumor size) had previously received bevacizumab.

### Correlatives

### VEGF and VEGFR2

Plasma VEGF and VEGFR2 levels were tested in 55 patient samples with time points at pre-dose, 24 hours post-dose, and week three. While samples were analyzed from some patients at all three time points, not all time points were available for each patient. Of the 55 patients analyzed, six experienced either SD≥6 months or PR.

Plasma VEGF levels increased in patients at 24 hours post-dose with an average percent increase of 251% (n=33, p=0.001 with Wilcoxon signed-rank test). Overall plasma VEGF levels significantly increased from baseline at the three week time point (n=26, average increase of 1214%, p<0.001 with Wilcoxon signed-rank test). No significant differences were observed between patients with SD≥6 months/PR and patients with PD or SD for less than 6 months when comparing baseline values (n=6 vs. n=47, average of 109.6pg/ml vs. 266.9pg/ml, p=0.54 with Mann-Whitney test).

Plasma levels of VEGFR2 increased slightly overall from pre-dose to 24 hours post-dose in the 33 patients who had blood samples available for both time points (average increase of 6.98%, p<0.001 with paired t-test). Another small overall increase in plasma VEGFR2 was observed from pre-dose to Week 3 (average increase of 10.19%, p=0.02 with paired t-test), although only 8 patients had samples at both time points. No significant differences were observed between patients with SD≥6 months/PR and PD/SD<6 months when comparing baseline values (n=6 vs. n=47, average of 8010pg/ml vs. 7844pg/ml, p=0.83 with unpaired t-test).

### HIF-1α

**Figure 3:**
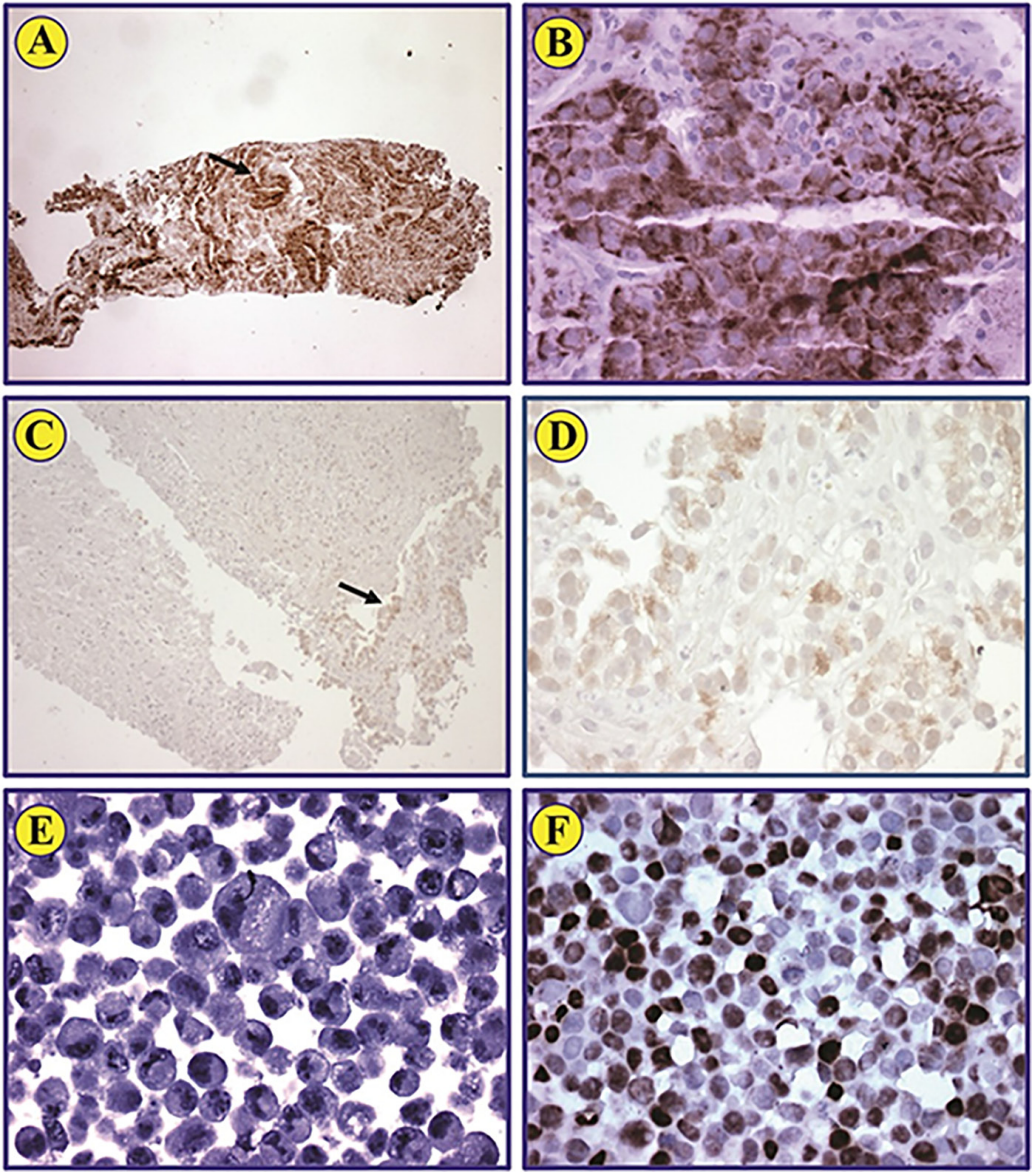
HIF-1α expression in pre- and post-treatment biopsies from renal cell carcinoma patient Immunohistochemical staining of a representative biopsy shows that HIF-1α protein is strongly expressed in renal cell carcinoma cells (A; original magnification: ×100). Neoplastic cells identified by the arrow are also illustrated at a higher magnification (B; ×400). Post-treatment biopsy from the same patient shows that scattered neoplastic cells that represent less than 1% of the biopsy express very weak levels of HIF-1α (C). The arrow identifies these cells (C), which are also shown at a higher magnification (D; ×400). Negative control MCF-7 cells lack the expression of HIF-1α (E; ×400). Positive control MCF-7 cells treated with CoCl_2_ demonstrate strong expression of HIF-1α (F; ×400).

Among the 14 patients who underwent tumor biopsies while on study, only five patients had adequate tissue from both the pre- and post-dose tumor biopsies. Characteristics of HIF-1α analysis in all biopsied tissue is displayed in Supplementary Table S1. Among the five patients with both pre- and post-dose tumor biopsies, four had positive HIF-1α staining at baseline. All four patients with positive HIF-1α at baseline demonstrated a decrease in HIF-1α staining in the post-dose biopsy, including three patients whose post-dose biopsy became completely negative for HIF-1α. The average percentage of cells staining for HIF-1α at baseline in these five patients was 68% (100%, 100%, 100%, 40%, and 0%, respectively) compared to 26% post-dose (40%, 0%, 0%, 0%, and 90%, respectively) (Figure 3). The small number of patients precluded statistical correlation with response, but two of the four patients with a decrease of HIF-1α expression experienced a decrease in tumor size (19% and 15% decrease in prostate and fallopian tube cancers, respectively).

### 20S Proteasome Activity

Inhibition of 20S proteasome activity was analyzed in plasma samples obtained from 31 patients. The figures can be viewed in Supplementary Figure S1. Three of the 31 patients experienced PR or SD≥6 months. Overall, 20S proteasome activity significantly decreased within 1 and 4 hours after treatment (average decrease of 10.44%, p=0.014 and 18.81%, p=0.004, respectively, with Wilcoxon signed-rank test), but changed inconsistently after day 2/3 (average increase of 3.54%, p=0.925 with Wilcoxon signed-rank test). This trend was observed whether the samples were separated by dose level (dose levels of bortezomib at 0.7-1.0mg/m^2^ (low) vs. 1.3mg/m^2^ (high)), response (SD≥6 months/PR vs. SD<6 months/PD), or toxicity (Grade 2 or greater thrombocytopenia, diarrhea, nausea, vomiting, and/or neuropathy vs. none/other). No significant differences in change in 20S proteasome activity were observed when comparing patients with SD≥6 months/PR against all others, when comparing patients with the described toxicities against all others and also stratified by dose, or when comparing low versus high dose levels. Some of these comparisons, such as those for SD≥6 months/PR against all others were limited by the small number of patients.

### DCE-MRI

All patients were screened for eligibility for DCE-MRI. The DCE-MRIs of 14 patients were analyzed. Among the 14 patients, 12 patients had DCE-MRI performed at all three time points (baseline, 24-48 hours, and end of cycle 1), and two patients had DCE-MRI performed at only two time points (baseline and 24-48 hours).

DCE-MRI analysis demonstrated a trend in dose-dependent decreases in *Ktrans* at 3 weeks (R=−0.83, p=0.0053) (Figure 2a). Patients who were treated at higher dose levels had a larger percentage decrease in *Ktrans* at 3 weeks. Standard error was calculated for each time point and is included in Figure 2a. No statistically significant dose-dependent trend was observed at the 24-48 hour time point.

Among the 14 patients evaluated, four patients received more than four cycles of treatment. These four patients did not demonstrate a statistically significant trend of greater decrease of *Ktrans* at either the 24-48 hour time point or the 3 week time point. At 24-48 hours, the decrease of *Ktrans* among patients who eventually received more than 4 cycles of treatment was 18.3% (standard error 21.2%), compared to 24.2% (standard error 48.0%) in the remainder of the patients. This trend of difference between the two groups was not statistically significant (p=0.24 with paired t-test). At 3 weeks, the decrease of *Ktrans* among patients who received more than 4 cycles of treatment was 14.4% (standard error 12.8%), compared to 16.4% (standard error 9.2%) in the remainder of patients (p=0.79 with two-tailed paired t-test). A representation of DCE-MRI analysis of a patient with nasopharyngeal carcinoma is shown in Figure 2b.

### VEGF selected genotypes analysis

Because of previous published evidence that polymorphisms of VEGF may correlate with the efficacy and toxicity of bevacizumab combination treatment, analyses of associations among selected VEGF genotypes and treatment outcomes were performed [35]. Schneider et al. previously demonstrated that VEGF-2578 AA and VEGF-1154A correlated with superior overall survival and VEGF-634 CC and VEGF-1498 TT associated with less grade 3/4 hypertension.

Patients (n=7) with VEGF-634 GG genotype compared to 9 patients with VEGF-634 GC and VEGF-634 CC genotypes had longer median time to treatment failure (TTF) (1.8, 95% CI 0.5-3.1 vs. 0.7 months, 95% CI 0.1-1.3; p=0.045) and patients (n=6) with VEGF-634 GC genotype compared to 10 patients with VEGF-634 GG and VEGF-634 CC genotypes had shorter median TTF (0.7, 95% CI 0.2-1.2 vs. 1.3 months, 95% CI 0.1-2.5; p=0.042).

Patients (n=4) with VEGF-2578 AA genotype compared to 20 patients with VEGF-2578 AC and VEGF-2578 CC genotypes had longer median overall survival (OS) (19.8 months, 95% CI 0.0-44.9 vs. 7.5 months, 95% CI 6.6-8.4; p=0.03). Also, 4 patients with VEGF-1498 CC genotype compared to 14 patients with VEGF-1498 CT and VEGF-1498 TT genotypes had longer median OS (21.8, 95% CI 0.0-46.9 vs. 7.5 months, 95% CI 4.2-10.8; p=0.019) and 11 patients with VEGF-1498 CT genotype compared to 7 patients with either VEGF-1498 CC or VEGF-1498 TT genotypes had shorter median OS (5.7 months, 95% CI 2.4-9.0 vs.21.7 months, 95% CI 0.0-44.3; p=0.023). Finally, 7 patients with VEGF-634 GG genotype compared to 9 patients with VEGF-634 GC and VEGF-634 CC genotypes had longer median OS (19.5, 95% CI 0.0-46.4 vs. 4.2 months, 95% CI 1.6-6.8; p=0.029) and 6 patients with VEGF-634 GC genotype compared to 10 patients with VEGF-634 GG and VEGF-634 CC genotypes had shorter median OS (2.4, 95% CI 0.0-5.9 vs. 12.9 months, 95% CI 0.0-29.2; p=0.008).

## DISCUSSION

In this study, we report the results of a phase I dose-escalation trial of combination bevacizumab and bortezomib. The rationale for this combination from preclinical and clinical studies was based on the use of bortezomib to overcome resistance to anti-angiogenic therapy via blockade of HIF-1α upregulation [3, 7-14, 19, 33]. The combination of drugs was well-tolerated. We successfully completed dose escalation to the highest specified dose level, i.e. the FDA-approved doses of both drugs (bevacizumab 15mg/kg and bortezomib 1.3mg/m^2^), without reaching a MTD. Therefore, the recommended phase 2 dose was determined to be the full FDA-approved doses for both drugs.

The safety profile of combination treatment with bevacizumab and bortezomib was successfully determined, with the caveat that the MTD was never reached in this study. Combination treatment with bevacizumab and bortezomib was well-tolerated, with 36% of patients who experienced no treatment-related toxicity greater than grade 1 and 72% of patients who experienced no treatment-related toxicity greater than grade 2. Treatment-related grade 3 and 4 toxicities included thrombocytopenia, fatigue, nausea/vomiting, diarrhea, neuropathy, anemia, neutropenia, and hypertension, which are consistent with the known toxicity profiles of the drugs. There were no treatment-related deaths.

Five of 20 evaluable patients with advanced renal cell carcinoma (median=4 prior treatments) experienced SD≥6 months/PR on study (PR; N=3). The number of patients is small but the 25% SD≥6 months/PR rate was higher than would have been expected for treatment with either agent alone in heavily pretreated patients [24, 36-38], suggesting that the combination may warrant further study in this histology. In phase II studies of renal cell carcinoma patients treated with bevacizumab in the first-line or second-line metastatic setting, response rates of 13% and 10%, respectively, were observed [24, 36]. In phase II studies of renal cell carcinoma patients treated with bortezomib in the second- or third-line setting, response rates of 5% and 11%, respectively, were observed [37, 38].

Two of six patients (33%) with nasopharyngeal carcinoma experienced SD≥6 months (N=1) or a PR (N=1) on study. No monotherapy studies of bevacizumab or bortezomib have evaluated activity in nasopharyngeal carcinoma, and there have been only limited combinations studies. In one previous study, 46 patients with stage IIB-IVB nasopharyngeal carcinoma were treated with bevacizumab in addition to standard chemoradiation. The two-year locoregional progression-free survival rate was 84% compared to 90% in other chemoradiation studies [39]. In a phase I dose escalation study of bortezomib with gemcitabine and liposomal doxorubicin, 1 nasopharyngeal carcinoma patient out of 11 patients with advanced head and neck cancers (9%) experienced a partial response [40]. A larger, biomarker-driven study may be warranted for combination bevacizumab and bortezomib in nasopharyngeal carcinoma to further explore the efficacy of this regimen.

HIF-1α has previously been shown to play an important role in renal cell carcinoma. In at least 60% of patients with sporadic clear-cell renal-cell carcinoma, the von Hippel–Lindau tumor-suppressor gene (VHL) is inactivated [41]. The VHL protein plays a critical role in the cellular pathway that couples changes in oxygen availability to gene expression through the regulation of HIF-1α. Under normal oxygen conditions, VHL binds HIF-1α, which leads to degradation of HIF-1α through various mechanisms. When VHL is not present, HIF-1α is not degraded. HIF-1α accumulates inappropriately in VHL-deficient cells during conditions of normal oxygen tension [41]. These cells overexpress HIF-regulated genes, including genes encoding angiogenic factors [41]. The upregulation of HIF in cells deficient in VHL is critically important in the tumorigenesis of renal cell carcinoma [42]. Inactivation of HIF can inhibit tumorigenesis among VHL-deficient renal carcinoma cells in xenograft models [43]. VHL inactivation results in increased activity of HIF and increased VEGF expression [42]. Inhibition of VEGF with bevacizumab and inhibition of HIF-1α by bortezomib is a logical therapeutic combination for the treatment of RCC.

In our study, HIF-1 expression was evaluated based on bortezomib's ability to downregulate HIF-1α in addition to proteasome inhibition. While the sample size was limited and few patients received paired biopsies, decreases in HIF-1α expression were observed in four or the five patients with paired biopsies, including two patients who experienced a decrease in tumor size (although not reaching PR). Future studies in larger numbers of patients would be needed to explore the relevance of HIF-1α expression as a potential predictive marker. Of note, mTOR inhibitor temsirolimus has also demonstrated evidence of HIF-1α inhibition, and a clinical trial combining temsirolimus with bevacizumab and liposomal doxorubicin demonstrated extensive anti-tumor activity in multiple tumor types [44].

Similar to previously published observations [45], no association between plasma VEGF or VEGFR2 changes was associated with response or duration of treatment. The small numbers of patients precludes robust calculations. New biomarkers need to be investigated for association with response to anti-angiogenic activity.

Consistent with the results of VEGF SNP analysis in the ECOG 2100 Phase III breast cancer trial [35], VEGF-2578 AA correlated with better overall survival compared to the AC and CC genotypes. However, the other significant associations identified in that study (VEGF-1154A with superior overall survival; VEGF-634 CC and VEGF-1498 TT associated with less grade 3/4 hypertension) were not replicated in our clinical trial, possibly because of the small sample size or because of the heterogeneity of our patient population.

Dynamic contrast-enhanced magnetic resonance imaging (DCE-MRI) performed at high temporal resolution following the administration of a gadolinium (Gd)-chelated contrast medium is a noninvasive imaging technology that can be used to measure properties of tissue microvasculature. DCE-MRI is sensitive to changes in blood volume and vascular permeability that can be associated with tumor angiogenesis, and consequently DCE-MRI is a promising biomarker for characterizing tumor response to anti-angiogenic treatment [46-49].

In our study, preliminary assessment of *K^trans^* on serial DCE-MRI demonstrated a trend of dose-dependent decrease of *K^trans^* at 3 weeks, suggesting that greater inhibition of angiogenic activity was achieved with higher doses of the study medications. Because of the limited number of patients who underwent evaluation with DCE-MRI, correlations between clinical response and change in DCE-MRI perfusion parameters are primarily exploratory in nature. Among the 14 patients evaluated, the four patients who received more than four cycles of treatment did not demonstrate a trend of greater decrease of *Ktrans* at either the 24-48 hour time point or the 3 week time point, compared to the remainder of the patients.

Overall, 20S proteasome activity significantly decreased within 1 and 4 hours after treatment (mean change −10.44%, p=0.007 and −18.81%, p=0.004, respectively), but changed inconsistently after Day 2/3. This trend was observed independent of dose level (dose levels of bortezomib at 0.7-1.0mg/m^2^ (low) vs. 1.3mg/m^2^ (high)), response (SD≥6 months/PR vs. SD<6 months/PD), or toxicity (grade 2 or greater thrombocytopenia, diarrhea, nausea, vomiting, and/or neuropathy vs. none/other). Contrary to expectations, pharmacodynamic inhibition of the 20S proteasome was not dose-dependent. Bortezomib's ability to inhibit 20S proteasome activity in a dose-dependent manner is well documented in the original dose-escalation trials of single-agent bortezomib. Potential drug-drug interaction between bortezomib and bevacizumab may be considered as a potential cause of the findings.

In conclusion, the combination of bevacizumab and bortezomib is well-tolerated at full FDA-approved doses of each drug, and has demonstrated clinical activity in patients with heavily pre-treated, advanced malignancy. Preliminary assessment of pharmacodynamic biomarkers and DCE-MRI suggests that inhibition of angiogenic activity was achieved. Because partial responses were observed, especially among patients with renal cell carcinoma who had previously progressed on anti-angiogenic treatment strategies, this treatment regimen merits further evaluation.

## MATERIALS AND METHODS

### Patients

Patient eligibility criteria included patients with advanced or metastatic cancer, who progressed following standard therapy or for whom no standard effective therapy is available; Eastern Cooperative Oncology Group (ECOG) performance status of ≤2; adequate bone marrow function (leukocytes ≥3,000/ml, absolute neutrophil count ≥1,500/ml, platelet count ≥75,000/ml), liver (bilirubin ≤2.0 mg/dL, alanine aminotransferase (ALT) ≤3x upper limit of normal (patients with liver metastases allowed bilirubin ≤3xULN, ALT ≤5xULN)), and kidney (creatinine ≤2xULN); and were at least 4 weeks beyond other chemotherapy or radiotherapy (at least 1 week if palliative low dose radiotherapy given to the limbs, at least 6 weeks with nitrosourea or mitomycin-C, or at least 5 half-lives or 4 weeks, whichever is shorter, for patients who received non-chemotherapeutic biologic agents) and recovered to Grade ≤1 toxicity. Patients with hemoptysis or clinically significant unexplained bleeding within 28 days of entering study, uncontrolled hypertension, clinically significant cardiovascular disease, uncontrolled intercurrent illness, hypersensitivity to study drug components, and pregnant/lactating women were excluded.

### Procedures

A modified 3+3 dose escalation scheme was used. Six patients were enrolled at each dose level. The cohort defined as the maximum tolerated dose (MTD) was permitted to be expanded by up to 10 patients to further evaluate toxicity and correlative data. At the MTD, up to an additional 10 patients with renal cell carcinomas (RCC) were permitted to be enrolled to further evaluate safety and efficacy. If a response was observed in a particular tumor type with the study drug or drug combination, then the study was permitted to be expanded to include a total of 14 participants with that tumor type. Up to an additional 15 patients with biopsiable disease were permitted to enroll at the MTD once it was determined, for the purpose of exploratory analysis with additional optional correlative studies. All enrolled participants were considered in the dose-limiting toxicity (DLT) analysis. Bevacizumab was infused intravenously on day 1 of each 21-day cycle with a dose escalation range of 2.5-15mg/kg. Bortezomib was infused intravenously on days 1, 4, 8, and 11 of each 21-day cycle with a dose escalation range of 0.7-1.3mg/m^2^. Bortezomib was infused only on days 1 and 8 of each cycle at the initial dose level. Treatment was repeated once every 21 days until prohibitive toxicity or intercurrent illness, tumor progression, or patient withdrawal.

Toxicities were graded using the Common Terminology Criteria for Adverse Events (version 3.0). Tumor response was assessed with Response Evaluation Criteria in Solid Tumors (RECIST) 1.0 and World Health Organization (WHO) criteria. Baseline radiological assessment was done within 28 days before starting treatment. Restaging evaluations were performed every 2 cycles (6 weeks).

### Correlatives

### Dynamic Contrast-Enhanced Magnetic Resonance Imaging (DCE-MRI)

Optional dynamic contrast-enhanced magnetic resonance imaging (DCE-MRI) was performed on consenting patients at the following time points: 1. baseline (within one week before day 1 treatment), 2. acute phase (24 - 48 hours after first infusion of bevacizumab and bortezomib), 3. chronic phase (at end of cycle 1). All MR data were acquired on a 1.5T Excite HD scanner (GE Healthcare, Waukesha, WI) with CRM gradient system. DCE-MRI was performed at high temporal resolution (<10s per imaging volume). Sequential magnetic resonance images were obtained before, during, and following the injection of gadolinium (Gd)-chelated contrast medium. Following the acquisition of localizer images, T1 mapping data were obtained using a multiple flip angle fast spoiled gradient recalled echo FSPGR sequence in a plane that included the target lesion(s) as well as a reference vessel. Following the T1 mapping acquisition, the DCE-MRI scans were obtained during slow steady breathing using the same pulse sequence and from the same acquisition volume before, during, and following bolus administration of 0.1mmol/kg gadopentetate dimeglumine (Magnevist, Bayer Healthcare) at 3ml/s followed by a 20ml saline flush also given at 3ml/s. The total DCE-MRI acquisition lasted about 8 minutes. All DCE-MRI data were analyzed using a two-compartment model to yield the pharmacokinetic parameters: endothelial transfer constant (*K^trans^*, min^−1^), extracellular extravascular space volume fraction (*v_e_*, unitless), and contrast agent reflux rate constant (*k_ep_*, min^−1^), using CineTool/Kinmod (GE Global Research Center/GE Healthcare) software developed in the IDL environment [50].

### Plasma Assessment of VEGF/VEGFR2

Optional whole blood (7 mLx2) was collected from consenting patients at the following time points: pre-dose baseline, 24-48 hours after the day 1 cycle infusion, and at the end of cycle 1. Plasma levels of VEGF and VEGFR2 in duplicate samples were evaluated using enzyme-linked immunosorbent assay (ELISA) (human VEGF QuantiGlo ELISA Kit recognizing VEGF121 and VEGF165 isoforms and human sVEGF R2/KDR Quantikine ELISA Kit, respectively) (R&D Systems, Minneapolis, MN) following the manufacturer's instructions (lower limit of detection=3.30pg/mL and 4.6pg/mL, respectively).

### Plasma Assessment of 20S Proteasome Inhibition

To determine proteasome 20S inhibition, optional whole blood (7 mLx2) was collected and PBMCs were isolated from consenting patients at the following time points: pre-dose baseline, and then 1, 4, and 24 hours after the initial bortezomib infusion on day 1 of cycle 1. After isolation, cells were washed with PBS, placed in two labeled cryovials and stored at −70°C in equal volume of RPMI-1640 with 20% DMSO until analysis.

Proteasome 20S inhibition was determined, in duplicate samples, by using a validated 20S proteasome activity EIA kit, APT280 (Chemicon, Temecula, CA) following the manufacturer's instructions (lower limit of detection measured with control assay was 20ng/100μl reaction).

### Immunohistochemical staining to assess the expression of HIF-1α in tumor biopsies

Optional tumor biopsies were assessed for evidence of malignancy on H&E stained slides. Immunohistochemical staining to detect the expression of HIF-1α protein was performed using standard techniques. Briefly, formalin-fixed and paraffin-embedded tissues were deparaffinized using alcohol gradient. Slides were subsequently washed in 3% H_2_O_2_ for 15 min to block endogenous peroxidase activity. Antigen retrieval was performed using 1× DAKO Target Retrieval Solution (DAKO, Carpinteria, CA) for 20min. Slides were then allowed to cool down for 20min at room temperature and blocked for 30min using the serum-free block solution in the Universal LSAB+ kit (DAKO). Purified mouse anti-human HIF-1α monoclonal antibody (catalogue number: 610958, BD Biosciences, San Diego, CA) diluted in blocking buffer (1:100) was added overnight at 4°C. Sequentially, slides were washed 3 times for 10min and incubated with secondary antibody LINK and then with secondary antibody Streptavidin each for 30 min. Slides were developed with 3,3´-diaminodbenzidine tetrahydrochloride substrate that includes horseradish peroxidase, and hematoxylin was used for counterstaining. MCF-7 breast cancer cells were either untreated or treated with CoCl_2_ for 4 h to induce the expression of HIF-1α and then used as negative or positive control, respectively. MCF-7 cells were processed in formalin to prepare paraffin embedded cell pellets before staining. Photomicrographs were captured using an Olympus DP70 camera (Olympus America, Melville, NY) and the QCapture Suite Plus software (Qimaging, Surrey, BC, Canada).

### VEGF Single Nucleotide Polymorphisms (SNPs)

DNA was extracted from peripheral blood mononucleocytes (PBMCs) or paraffin-embedded tissue sections using the QIAamp^®^ DNA Mini and Blood Mini Kit or QIAamp^®^ DNA FFPE Tissue Kit (Qiagen, Valencia, CA) according to standard protocols recommended by the manufacturer. The region of interest was then amplified using custom PCR primers. Sanger sequencing was performed on a 3730xl DNA Analyzer (Applied Biosystems) using BigDye™ Terminator v3 chemistry (Applied Biosystems). Mutation analysis was performed using SeqScape® Software v2.5 (Applied Biosystems).

### Statistical Analysis

Analyses were descriptive and exploratory. Within-patient comparisons were analyzed using paired t-tests and Wilcoxon signed rank tests. Between-patient comparisons were analyzed using unpaired t-tests and Mann-Whitney tests. Correlations were assessed using either Pearson or Spearman correlation analyses. Non-parametric analyses were chosen when data were clearly not normally distributed and/or had clear outliers. Analysis of VEGF SNPs was performed using SPSS 19 computer software (SPSS Chicago, IL). Many of the analyses were based on small numbers of patients, and care must be taken when interpreting non-statistically-significant results. Due to the exploratory nature of the analyses, no adjustment was made for multiple testing.

## SUPPLEMENTARY DATA


