# Supplementary material for: Targeting hypoxia-inducible factor-1α (HIF-1α) in combination with antiangiogenic therapy: A phase I trial of bortezomib plus bevacizumab
Source: Oncotarget. 2014 Nov 8;5(21):10280–92. doi: 10.18632/oncotarget.2163 (PMC4279372; doi:10.18632/oncotarget.2163)
Supplement: Supplementary file 1 [file oncotarget-05-10280-s001.pdf]

SUPPLEMENTARY DATA

Table S1: HIF-1α expression before and after treatment

Patient Information			Predose Biopsy				Post-Dose Biopsy			
Diagnosis	Best Response	Months on Study	HIF1	% positive	Intensity	Location	HIF1	% posi-tive	Intensity	Location
Nasopharyngeal	SD (–21%)	6	No tissue in biopsy sample				+	100%	½	cytoplasm
Prostate	SD (–19%)	5	+	100%	3	cytoplasm	+	40%	2	cytoplasm + nucleus
Fallopian Tube	SD (–15%)	8	+	100%	2	cytoplasm	-	n/a	n/a	n/a
Granular Cell Carcinoma	SD (–8%)	5	No tissue in biopsy sample				-	n/a	n/a	n/a
GE junction	SD (5%)	2	Tissue block could not be located				-	n/a	n/a	n/a
Bladder	Clinical PD	1	+	70%	3	cytoplasm	Biopsy not performed			
Colorectal	Clinical PD	2	+	100%	½	cytoplasm	Biopsy not performed			
Paraganglioma	Clinical PD	< 1	-	n/a	n/a	n/a	Biopsy not performed			
Renal Cell	Clinical PD	1	+	100%	3	cytoplasm	-	n/a	n/a	n/a
Prostate	PD (23%)	1	+	40%	½	cytoplasm + nucleus	-	n/a	n/a	n/a
Melanoma	PD (38%)	2	+	90%	Variable (1-3)	cytoplasm	Tissue block could not be located			
SCC H&N	Inevaluable (PD)	< 1	+	100%	3+	cytoplasm	Biopsy not performed			
Rectal	Inevaluable	< 1	+	100%	½	cytoplasm	Biopsy not performed			
Renal Cell Carcinoma	Inevaluable	1	-	n/a	n/a	n/a	+	90%	2+	cytoplasm

"+" = positive, "–" = negative
Intensity measured on a scale of 1-3
gray box = no sample

a.

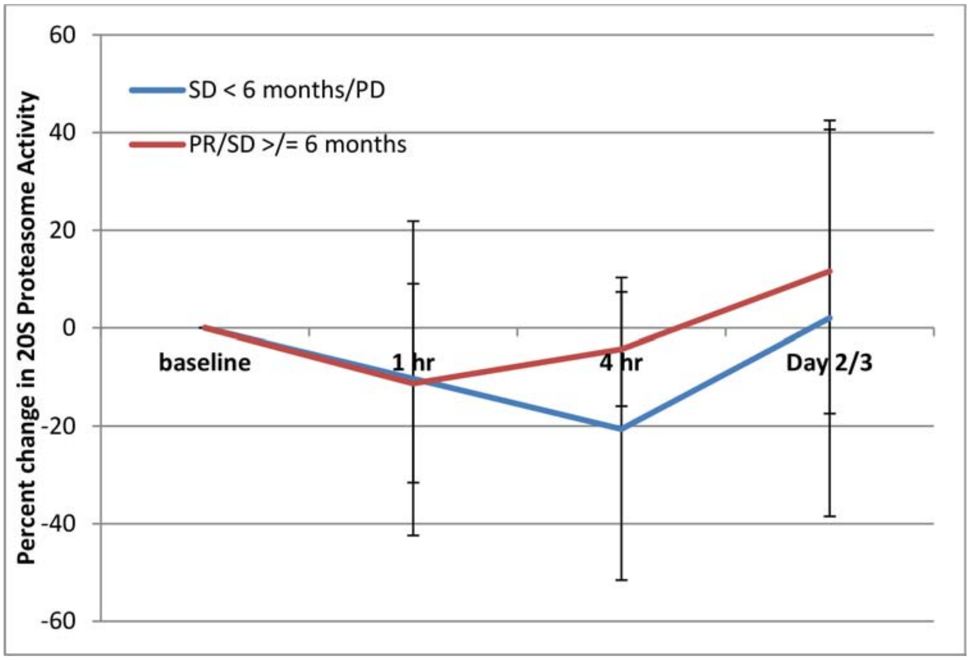

b.

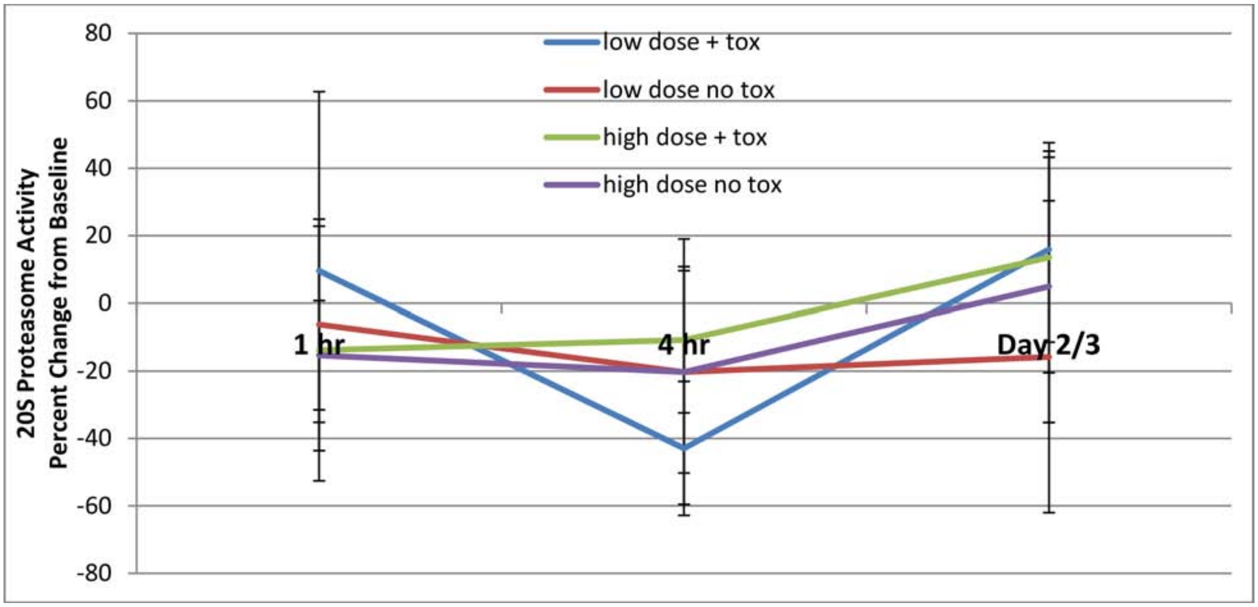

Figure S1: 20S Proteasome Inhibition. (**a**). **Mean change in 20S proteasome activity stratified by response.** Percent change in 20S proteasome activity from 31 patients was analyzed. Patients with PR or SD ≥ 6 months (n = 3) were analyzed in one group (red line) while patients with SD < 6 months or PD (n = 28) were analyzed in a second group (blue line). (**b**). **Mean change in 20S proteasome activity stratified by high/low dose and +/− toxicity.** Percent change in 20S proteasome activity from 31 patients was analyzed. Patients were stratified by dose levels and then further stratified by specific toxicities experienced (Grade 2 or greater thrombocytopenia, diarrhea, nausea, vomiting, and/or neuropathy vs. other). Eight patients received lower dose levels of bortezomib of 0.7-1.0 mg/m^2 and did not experience specified Grade ≥2 toxicities (red line) while three patients received lower dose levels of bortezomib and did experience Grade ≥2 toxicities (blue line). Nine patients received higher dose levels of bortezomib of 1.3 mg/m^2 and did not experience specified Grade ≥2 toxicities (purple line) while 11 patients received higher dose levels of bortezomib and did experience Grade ≥2 toxicities (green line).
